# A Service Brokering and Recommendation Mechanism for Better Selecting Cloud Services

**DOI:** 10.1371/journal.pone.0105297

**Published:** 2014-08-29

**Authors:** Zhipeng Gui, Chaowei Yang, Jizhe Xia, Qunying Huang, Kai Liu, Zhenlong Li, Manzhu Yu, Min Sun, Nanyin Zhou, Baoxuan Jin

**Affiliations:** 1 NSF Spatiotemporal Innovation Center, George Mason University, Fairfax, Virginia, United States of America; 2 School of Remote Sensing and Information Engineering, Wuhan University, Wuhan, Hubei Province, China; University of Vigo, Spain

## Abstract

Cloud computing is becoming the new generation computing infrastructure, and many cloud vendors provide different types of cloud services. How to choose the best cloud services for specific applications is very challenging. Addressing this challenge requires balancing multiple factors, such as business demands, technologies, policies and preferences in addition to the computing requirements. This paper recommends a mechanism for selecting the best public cloud service at the levels of Infrastructure as a Service (IaaS) and Platform as a Service (PaaS). A systematic framework and associated workflow include cloud service filtration, solution generation, evaluation, and selection of public cloud services. Specifically, we propose the following: a hierarchical information model for integrating heterogeneous cloud information from different providers and a corresponding cloud information collecting mechanism; a cloud service classification model for categorizing and filtering cloud services and an application requirement schema for providing rules for creating application-specific configuration solutions; and a preference-aware solution evaluation mode for evaluating and recommending solutions according to the preferences of application providers. To test the proposed framework and methodologies, a cloud service advisory tool prototype was developed after which relevant experiments were conducted. The results show that the proposed system collects/updates/records the cloud information from multiple mainstream public cloud services in real-time, generates feasible cloud configuration solutions according to user specifications and acceptable cost predication, assesses solutions from multiple aspects (e.g., computing capability, potential cost and Service Level Agreement, SLA) and offers rational recommendations based on user preferences and practical cloud provisioning; and visually presents and compares solutions through an interactive web Graphical User Interface (GUI).

## Introduction

As a new computing paradigm, cloud computing provides the capability of delivering elastic and virtually unlimited computing capacity as the 5^th^ utility [1]. The proliferation of cloud computing technologies is exemplified by the number of cloud vendors and their services, has produced numerous options for cloud users, and at the same time brings the complexities and challenges for selecting cloud service.

Applications and scientific research benefit from the virtually unlimited resources of cloud computing to meet the increasingly complicated and challenging requirements for computing resources [2]–[5]. Public cloud services provide convenience, including reducing the initial time investment and learning curve for building cloud solutions for users with limited knowledge on cloud computing. However, it is still a challenge to select the most suitable solution to deploy and configure applications due to the following reasons:

- **Application requirements**: different application features (e.g., data volume, data production rate, data transfer and updating, communication, computing intensities) result in varying computational intensity (e.g., data intensity, computing intensity, communication intensity) and disparate computing resource requirements (e.g., CPU, memory, storage, and network bandwidth).
- **Business expectations**: applications and potential users of the applications differ, which result in different budget investments (fee constraints) and expectations of cloud services. Meanwhile, various pricing models (e.g., on demand/reserved/bidding mode), pricing items (e.g., VM, dedicated server, storage, IP, network, software packages, custom services) and business strategies further complicate the selection.
- **Capacity provisioning**: commercial and open-source cloud services adopt different IT technologies (e.g., virtualization, storage) and have unique strengths and weaknesses. The learning curve to fully understand these technologies is steep.
- **Cloud information collection and process**: to compare cloud services, users ”march” through multiple websites individually to collect the required information and conduct assessments manually (e.g., cost analysis). This manual process is time-consuming.

The interweaving of these factors makes cloud service selection problematic. It is not only a technical issue but a decision-making problem, involving trade-offs among business expectations, investment cost, capacity provisioning, application requirements, rules and policies [6]. Making a sensible and correct decision is not easy for all levels of experienced users as applications, options and platforms can vary significantly.

There is valuable research on cloud service measurement, simulation, evaluation, brokering, and state-of-the-art cloud advisory systems. However, for specific applications, there is no comprehensive research integrating application preferences/constraints, computational features, and real-world cloud resource provisioning to assist with the generation, comparison and recommendation of cloud solutions. This research proposes a brokering and recommendation mechanism coupled with a corresponding tool to assist users to compare and select cloud solutions. Such a system leverages the following capabilities: 1) automatically collect heterogeneous cloud information from different cloud services and depict a uniform information model; 2) generate specific configuration solutions by aggregating different cloud resources for target applications (i.e., cloud solutions) based on users' preferences and constraints; 3) evaluate and recommend cloud solutions by leveraging multiple selection criteria (e.g., potential cost and the fitness upon computational requirements and features). Based on such a mechanism, a cloud advisory tool with integrated computing experiences and knowledge is capable of recommending cloud solutions for achieving both cost-efficiency and high performance. Each of these cardinal features is the basis for Section 1 of this paper. The remainder is organized as follows: Section 2 reviews related work; Section 3 introduces the methodologies and system architecture; Section 4 reports the implementation of the prototypes, experiment and results; and Section 5 concludes the paper and discusses future research and development.

## Related Work

### 2.1 Cloud Service Measurement, Simulation and Evaluation

Cloud metrics (http://collaborate.nist.gov/twiki-cloud-computing/bin/view/CloudComputing/RATax_CloudMetrics) is the foundation for cloud measurements, evaluation, and selection to assist cloud consumers to compare and understand the advantages and disadvantages of cloud services. Research has been conducted on establishing cloud metrics [7]–[9] and metrics-based evaluation algorithms [10], [11]. Specifically, Repschläger et al. [8] proposed a provider independent classification model with six target dimensions to compare IaaS providers. Martens et al. [9] summarized a maturity evaluation model for cloud services with nine different characteristics (e.g., SLAs, scalability, auditability, security). The degree of maturity is calculated using the weighted average of the criteria. National Institute for Standards and Technology (NIST) cloud Reference Architecture and Taxonomy Working Group (RATax WG) created a Cloud Metrics Sub Group to explore open issues and establish a consistent and operable measurement to enable stakeholders to communicate efficiently by standardizing the criteria and associated models [13]. The Cloud Service Measurement Initiative Consortium (CSMIC, http://csmic.org/) developed the Service Measurement Index (SMI) to define global measures for cloud service [14]. Despite progress, further efforts are needed to define consistent, reusable, and operational models to support comprehensive and objective measurements of cloud services [15].

A performance and application-based mechanism is best to evaluate open-source cloud solutions and commercial cloud services [16], [17]. CloudCmp [18] is a performance comparison tool developed to measure elastic computing, persistent storage and networking services. To investigate the readiness of public cloud services for supporting scientific computing, Jackson et al. [19] tested the performance of Amazon web services using eight selected high performance computing applications. Iosup et al. [20] conducted many-tasks performance analysis on four services of Amazon EC2, GoGrid, ElasticHosts and Mosso. Farley et al. [21] investigated the performance heterogeneity of supposedly identical instances and explored heterogeneity-aware placement strategies to find better-performing instances. Huang et al. [22] evaluated the readiness of open-source cloud solutions for supporting geospatial applications. The basic features and performances of three open source cloud software (i.e., OpenNebula, Eucalyputus and CloudStack) were compared in terms of cloud resource operation and geoscience application [22].

Simulation provides theoretical approaches to assist cloud service selection. CloudSim [23] is a toolkit to model and simulate a cloud computing environment. The toolkit provides a repeatable, controllable and cost-free environment for cloud customers to test their applications. CloudMIG [24], [25] is a simulation-based environment and user interface to evaluate cloud deployment options for supporting cloud migration. CloudMIG compares cloud service solutions and checks conformance and simulate workloads for envisioned cloud-based target architectures. Moreover, a genetic algorithm to optimize software deployment and reconfiguration rules is proposed [26] based upon CloudMIG, which relies on architecture analysis of target software. In order to capture code structures and their dependencies, and to check potential constraints violation in cloud environment, all source codes and dependent libraries must be imported and analyzed in CloudMIG. The code analysis process is time-consuming and introduces code privacy issues.

To help end users understand their Return On Investment (ROI), cost models were developed to estimate potential cost composition and utilization of imbalanced factors. Li et al. [27] developed an amortization and utilization models to calculate cloud Total Cost of Ownership (TCO) and Utilization Cost, respectively. Andrzejak [28] proposed a probabilistic model for optimizing monetary costs, performance and reliability given application requirements and dynamic conditions. The model helps consumers bid optimally on Amazon EC2 Spot Instances (http://aws.amazon.com/ec2/spot-instances/). A dynamic resource allocation algorithm [29] based on Model Predictive Control (MPC) best matches cloud customer demand with supply and price. The method maximizes the provider's ROI while minimizing energy cost.

The research provides theoretical methods to measure, simulate and evaluate cloud services. More specifically, the cloud metrics comprehensively measures cloud service. Simulation is a mechanism to study clouds' behaviors, capacities and status.

Performance analysis compares cloud solutions in a quantitative manner but rarely consider user preferences, constraints and computational features. To address this problem, we designed an operational service classification model for comparing different services and propose an application requirement schema to represent application-specific requirements and user preferences. However, there is no comprehensive consideration of application owner's preferences/constraints, computational feature of target applications and real-world cloud resource provisioning. The mechanism to integrate these methodologies for assisting generation, comparison and recommendation of cloud configuration solutions has not been systematically investigated.

### 2.2 Cloud Brokering Mechanism

Current cloud offerings have the following deficiencies [30]: limited scalability of single cloud service; lack of interoperability among cloud services; and no built-in business service management support. The brokering approach efficiently mediates, advertises and integrates heterogeneous cloud information and resources from different cloud providers.

The Reservoir model [30] draws a blue-print to open federate cloud services. In this architecture, cloud providers dynamically partner with each other to create an infinite IT resource pool while preserving their individual autonomy in making technological and business management decisions. Buyya et al. [31] proposed the utility-oriented federal cloud computing environment InterCloud, supporting auto-scaling across multiple vendor clouds. InterCloud dynamically expands and contracts to handle sudden changes in service demands. It consistently achieves QoS targets under various workload, resource and network conditions. To achieve a federate cloud framework still requires further development of tightly-coupled mechanisms and agreements to manipulate and integrate resources from multiple providers.

Goscinski and Brock [32] proposed a systematic method to publish, discover and select cluster resources by establishing a Resources Via Web Service framework (RVWS) using Service-Oriented Architecture (SOA). Under this framework, a cluster is encapsulated as a stateful web service using Web Service Resource Framework (WSRF) and is published through a dynamic discovery broker. The client queries and selects matched cluster based on resource states (e.g., free disk, free memory, CPU usage) and cluster characteristics (e.g., core number, core speed, hardware architecture). This framework provides an efficient method to monitor and manipulate the cluster through a web service interface. Although the proposed method is extendable to other cloud resources, most public cloud services do not yet provide such a mechanism to publicly describe and publish their resource offerings for discovery and selection. Furthermore, the real-time states of computing infrastructures may be non-transparent to the public [33].

These brokering mechanisms and federation frameworks mediates and integrates cloud services. However, the interoperability environment is too immature to achieve seamless and flexible integration. The specifications and initiatives for advertising, mediating, manipulating and orchestrating cloud services are at early stage. As such, declaring stateless resource provisioning through web pages/APIs is the primary channel to share cloud information. The method to efficiently collect and fuse cloud information from heterogeneous sources is a practical and critical. We propose a unified cloud information model and related collecting methods.

### 2.3 State-of-the-Art Cloud Advisory System

Cloud advisory websites simplify the search and comparison of cloud service by integrating information through one-stop portals. Service filtering and user reviews are two widely-supported basic functions. The former uses a feature-based (e.g., technology characteristics) filtering function to facilitate service matching, while the latter provides an approach to collect user feedback and conduct experience-based ratings. Data Center Map (http://www.datacentermap.com/cloud.html) combines a map-based visualization function and feature-based filter function. The map view visualizes the geographical distribution of data centers and IaaS cloud servers through Google Maps, where users search resources in certain regions, browse profiles, grade and conduct reviews. FindTheBest (http://cloud-computing.findthebest.com/) provides purchasing guidance and explains basic factors and concepts in cloud service selection. Furthermore, it personalizes ranking rules by allowing users to specify the important factors (e.g., compatible OS, control interface, support features, cost).

Third-party auditing provides a trustable understanding of the performance, reliability, and consistency of cloud services. Global Provider Viewer (https://cloudsleuth.net/global-provider-view) is a web-based tool for collecting and visually analyzing the performance and availability of PaaS and IaaS in a near real-time manner. It continuously monitors the top cloud services globally *via* Internet backbone locations by running a sample web application for each of the cloud services. The response time and availability of cloud services are analyzed at multiple geographical (e.g., global, continental, regional, city) and time (e.g., hours, days, months) scales. To better support visual analytics, multiple display methods (e.g., map view, linear series diagram, data tables) are integrated through web Rich Internet Application (RIA) technologies. The adopted quality monitoring method is proper for typical web applications. In order to comprehensively evaluate cloud services for different applications (e.g., scientific computing, business transactions), an elaborate auditing and monitoring architecture needs to be designed.

The developments of cloud service monitoring tools and advisory websites facilitate the measurement and comparison of cloud services. Besides collecting cloud information and visually comparing cloud services, an advisory system should generate application-specific configuration solutions upon real cloud provisioning and provide recommendations on specific application requirements and user preferences. To address this need, we developed a preference-aware solution evaluation model. The model predicts cost, measures VM computational capability and conducts an overall evaluation based on the importance of selected criteria.

In summary and to assist cloud service selection, a systematic brokering and recommendation framework is needed. This framework should integrate cloud information from multiple cloud providers, and create/evaluate solutions based on the application owners' requirements. We propose such a framework and introduce the corresponding models, methods and architecture. Instead of assessing cloud at service level, this research focuses on concrete configuration solutions (finer granularity) for applications upon application owners' requirements and real public cloud service (both IaaS and PaaS) resource offerings. The prototype generates feasible solutions, calculates potential cost, and evaluates/makes recommendations based on proposed models. The solutions give valuable references for specific applications in cloud adoption. The proposed framework and methods provide conceptual guidelines for designing and developing relevant advisory systems.

## Methodologies & System Architecture

**Figure 1:**
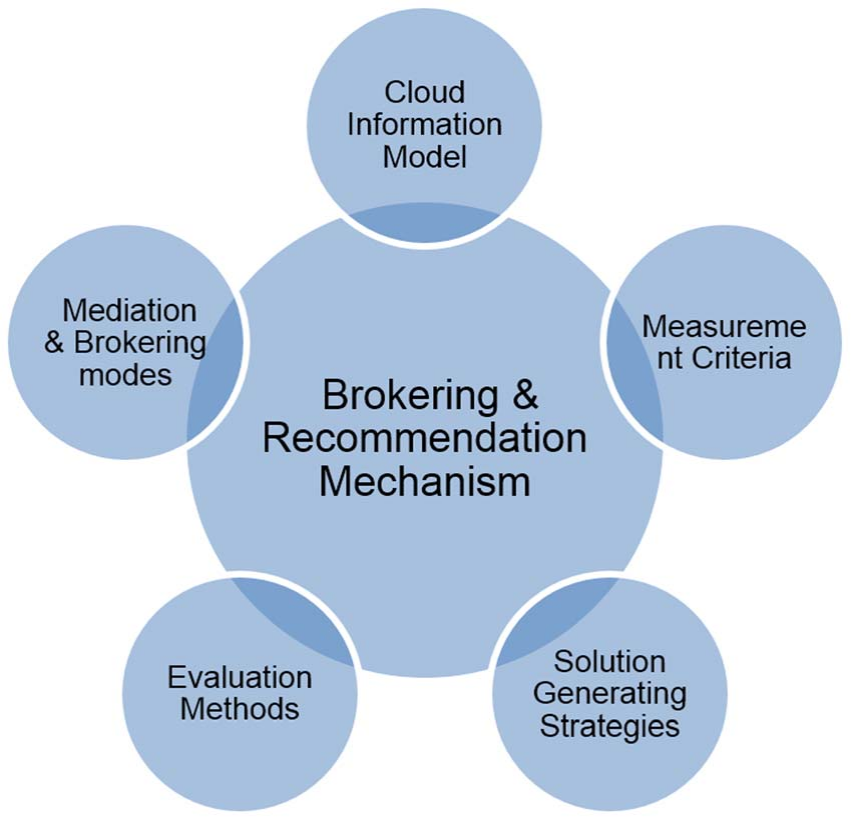
Building blocks of cloud brokering and recommendation mechanism.

To establish a systematic recommendation mechanism and to develop advisory systems sequentially, sophisticated methods and technologies should be developed (Fig. 1) that have the five charcateristics First, a unified cloud information model is essential to synthesize and depict heterogeneous cloud information. Second, mediation and brokering modes provide approaches to mediate and integrate information from different sources (e.g., cloud providers, auditors, consumers). Third, a model of measurement criteria facilitates solution filtering and evaluation by measuring capabilities of cloud services/solutions in qualitative and quantitive terms. Four, solution strategies determine modes and rules to generate solutions. And five, evaluation methods provide effective and preference-aware approaches to rank cloud solutions according to users' requirements.

### 3.1 A Unified Cloud Information Model

To evaluate cloud solutions, information needs to be fused, including resource offerings and pricing rules published by cloud providers, performance monitored by third-party audits and feedback from cloud users. The information originates from heterogeneous sources and is expressed differently. Even for widely-used public cloud services, different terminologies and expressions describe their provisioning, so a uniform and sophisticated information model is essential to manage heterogeneous information.

**Figure 2:**
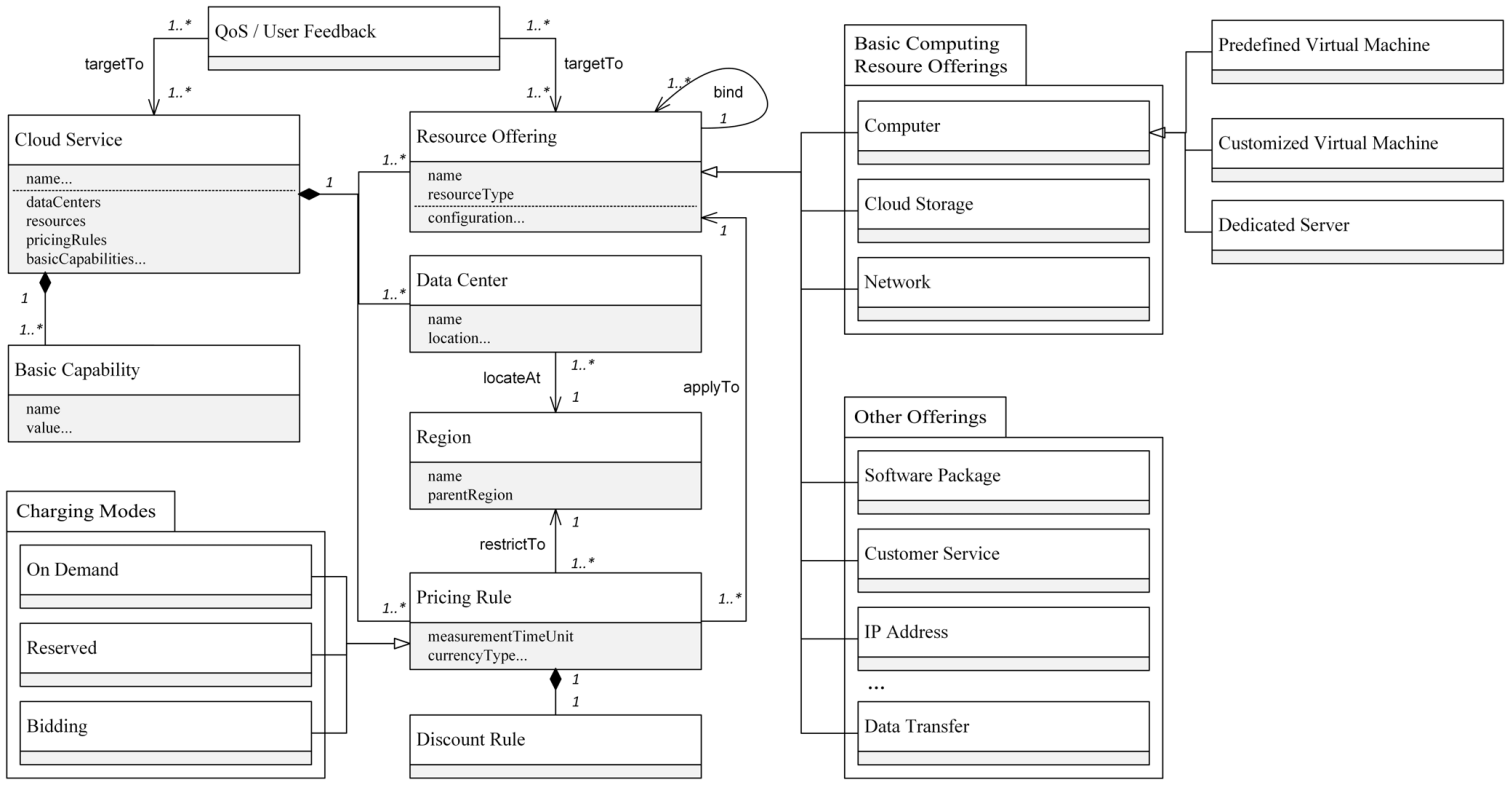
High-level hierarchy of cloud information model.

By generalizing information provided by ten public cloud services (section 4.3), audit performance and user feedback for cloud solution generation and evaluation, we propose a cloud information mode whose high-level hierarchy is illustrated (Fig.2). The top level element is Cloud Service, a service provided by a specific vendor. A *Cloud Service* is composed of four components: *Data Center*, *Basic Capability*, *Resource Offering* and *Pricing Rule*. The *Data Centers* are the physical allocation of Cloud Infrastructure and is an indicator for applications which need to meet geo-location related policy restrictions by avoiding cross boundary issues. *Basic Capabilities* describe technical (e.g., VLAN, data encryption, hypervisor type) or business modes (e.g., reserved or bidding pricing) that determine if the service is supported. These indicators can be used as filtering criteria. A *Resource Offering* depicts a physical/virtual resource, function or service provided by a cloud provider, which may be charged by usage or not at all. It is divided into basic computing resource offerings (i.e., computer, cloud storage, network) and complementary offerings (e.g., software package, OS template, customer service, snapshot/imaging/data backup/data transfer functions). To support different charging modes, the model defines three sub-types of Pricing Rules (i.e., on demand, reserved bidding modes). A *Pricing Rule* specifies a charging mode for a certain resource offering in a certain region because charging modes are geo-location related. Each rule has an associated measurement time unit and currency type. A resource offering may provide multiple charging modes. To support different charging modes, the model defines three sub-types of Pricing Rules (i.e., on demand, reserved and bidding modes).

Association relations are significant in this mode for several reasons. First, the Data Center and pricing rule are associated with Regional object as they are location related. Second, third-party Quality of Service (QoS) and user feedback are linked with cloud services or concrete resource offerings instead of being added as list properties. This makes the connection more flexible since the QoS and feedback are user-specific and changing frequently. Third, the setting of pricing rules and resource offering modes depends on the providers' business strategies, technical status and specific application demands, and different cloud services have different settings. For instances, services may offer multiple resources in batches as binding resources, whereas others offer them separately. To address this problem, the model describes basic Resource Offerings at an atomic resource level allowing them to be combined with each other through association relations. In summary, through an extendable hierarchical structure and associations among components, the information model is used for multiple cloud services.

**Figure 3:**
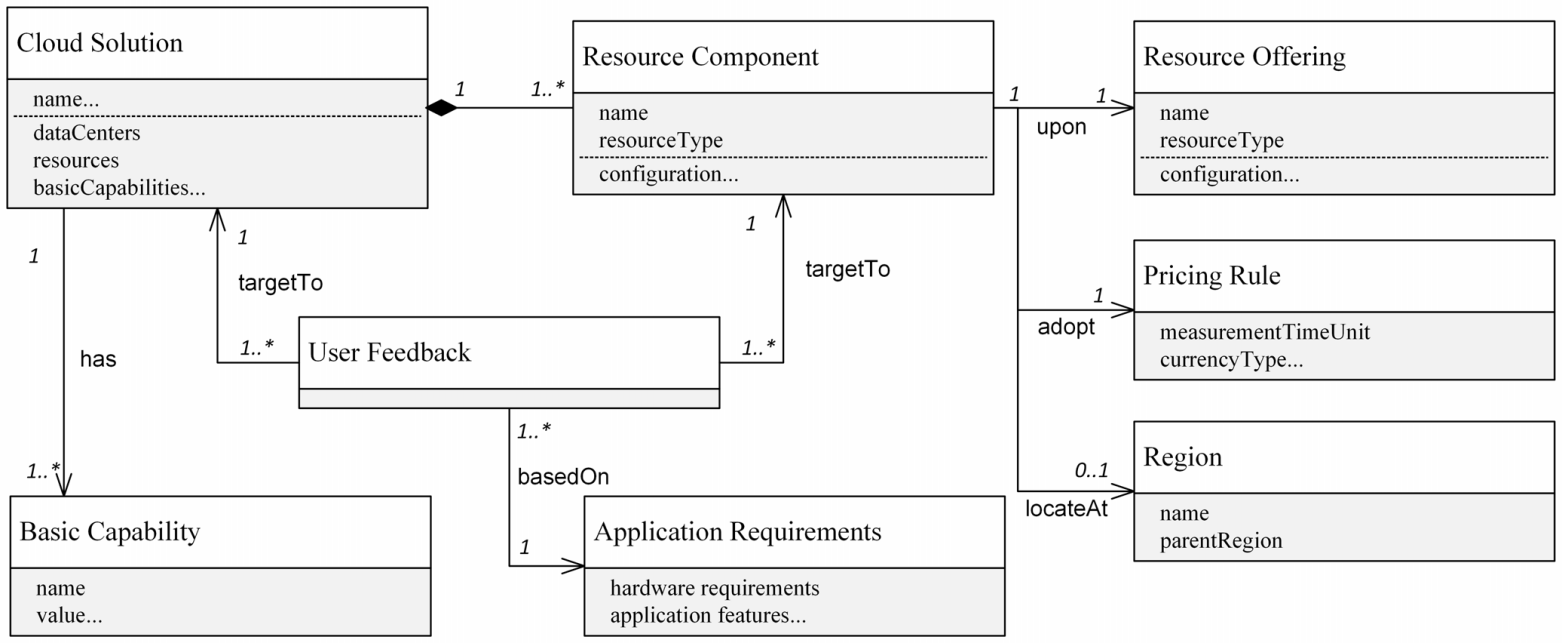
Structure of cloud solution.

Based on the above model, a cloud solution that users could adopt for their applications is expressed as a composition of multiple resource components (Fig. 3). A resource component is a resource offering at a certain region and with certain pricing rules. Resource components of a single solution can be provided by different services (e.g., hybrid solution in which cloud storage is envisioned by different providers). A solution can inherit basic capabilities from resource providers and the resourcing offerings.

### 3.2 Collecting and Managing Cloud Service Information

Collecting and managing up-to-date and heterogeneous cloud information from different cloud providers are critical for a cloud service recommendation system.

#### 3.2.1 Collecting Cloud Service Information

**Figure 4:**
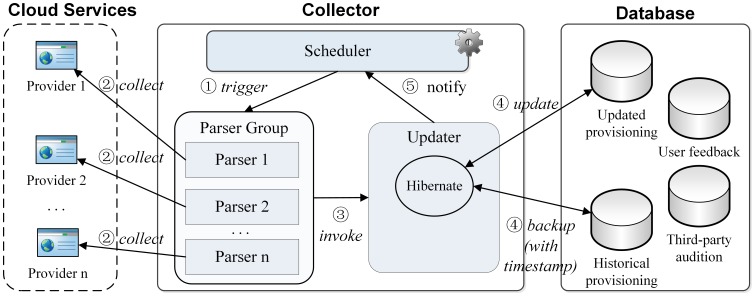
Structure and updating workflow of cloud collector.

Normally, the pricing rules and resource offerings of cloud services are frequently adjusted and published on the cloud providers' websites. Only a few providers offer web service-based APIs and specifications to the public. To automatically collect the information in a near real-time manner, we adopt a combined strategy of web page parsing and web APIs invocation. A proposed structure and interactions of an information collector are illustrated in Figure 4. A scheduler triggers update events on a daily basis and invokes the parsers in parser group. The parsers collect information through either web page parsing or web APIs invocation. For each cloud service, a dedicated parser translates collected information into entities and relations in the cloud information model. An updater inserts collected information into the database.

#### 3.2.2 Storage and Update of Cloud Information

The database contains four components (Fig. 4) as follows: user feedback for recording user reviews and grades; third-party audition for storing the performance summaries from auditors; updated provisioning for storing the latest information collected from cloud providers; and historical provisioning for recording every change in cloud provisioning for further analysis. To avoid redundancy, only changed atomic items are updated. For example, once the on-demand tenant price of a certain VM type changes, the price of related *Pricing Rule* in update provisioning is updated, after which a new *Pricing Rule* record associated with updating timestamp is inserted into historical provisioning. To avoid complex SQL operation on inserting, updating and retrieving, Hibernate is used to manipulate the information model and database.

### 3.3 Service Filtering Using an Operable Service Classification Model

**Figure 5:**
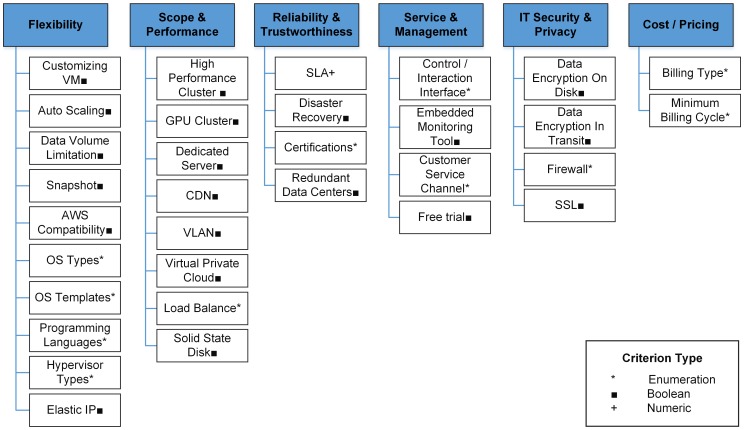
Structure of service classification model.

The service classification model are criteria organized with hierarchical tree structures to measure capabilities, limits and offerings of cloud services in logical and/or operable criterion layers (Fig. 5). The logical layer is a series of dimensions which describes capabilities demanded conceptually and which contain operational criteria. An operational criterion corresponds to a measureable and comparable atomic indicator describing a service-level feature of the cloud provider independently from any concrete service offering (e.g., existing certifications and IT infrastructure characteristics).

Six dimensions of the model (Fig. 3) based on relevant cloud metrics [7], [8], [14] were selected. First flexibility is a cardinal feature of cloud service, having the advantage of agility and scalability compared to traditional solutions. Within this characteristic, ten criteria are used for standardization of API, fertility of selectably predefined OS and software, customization of hardware and software, and scalability of capability offering. Second, *scope and performance* measures computational performance using a group of hardware/software criteria. Three, reliability and trustworthiness define how certain the customer is served as promised by the cloud provider. Trustworthiness is measured *via* provider's infrastructure features (e.g., disaster recovery, certifications, redundant sites). Four, service and management addresses features that determine the convenience of cloud service usages, including types and friendliness of user interfaces, auxiliary service options (e.g., monitoring, reporting) and customer service channels. Five, IT security and privacy are security related technical indicators. Six, cost/price addresses monetary consideration, including billing and penalty models.

These dimensions cover the most important aspects of cloud measurement, and the criteria are from well-accepted technical concepts [7], [8], [10], [12]–[14] and popular cloud services (section 4.3.1). Most criteria are qualitative, indicating whether a function/capability is supported or which types it supports. Thus, users with some cloud experiences can specify their service filtering rules. In addition, the model is not limited to the selected dimensions and criteria but can be expanded. Using these criteria, cloud providers are compared, classified and filtered. The model guarantees that the filtered services meet the user's demands at the service level. Meanwhile, service filtering makes cloud solution generation (next step) more efficiently since solutions are generated through qualified services only instead of all services.

### 3.4 Solution Generating with an Application-Dependent Requirement Description Schema

After selecting preferred and qualified cloud service, generating configuration and deployment solutions are the next task. So the generated cloud solutions match the requirements of specific applications, an elaborate requirement description at the application level is needed. We proposed an application-dependent requirement description schema (Table 1) consisting of three hardware requirements, application features and payment preferences.

**Table 1:** Parameters of application requirement schema.

Category/Source	Category Item	Description	Value Expression
**Hardware Requirements/User specified or Predefined default values according to application type**	VM Number +	expected machine number	<min, prefer>
	RAM +	physical memory size per machine (GB)	<min, prefer>
	CPU Core +	core number per machine	<min, prefer>
	CPU Speed +	speed of each core (GHz)	<min, prefer>
	Local Disk Size +	physical storage space per machine (GB)	<min, prefer>
	Bandwidth +	expected network speed (Mb/s)	<min, prefer>
	VM GeoLocation *	machine location constraint/preference	Region_ID
	Cloud Storage GeoLocation *	cloud storage location constraint/preference	Region_ID
	Hybird Solution ▪	whether cloud resources can come from different providers	true/false
**Application Features/User specified or generated according to intensity features**	Application Type *	types of the target application	AppType_ID
	OS Type *	required operating system to run, the application	OS_ID
	Application Size +	application size (after extra disk space) installation/deployment)	<µ, σ> or<µ, min, max>
	Extra Local Disk Size +	local data and switching space	<µ, σ> or<µ, min, max>
	VM Ingress Traffic +	ingress speed for each (virtual) machine (Mbps)	<µ, σ> or<µ, min, max>
	VM Egress Traffic +	egress speed for each (virtual) machine (Mbps)	<µ, σ> or<µ, min, max>
	Traffic Among VMs +	communication speed between machines	< µ, σ > or< µ, min, max>
	Concurrent Access Number +	concurrent access number to the application	<µ, σ> or<µ, min, max>
	Use Cloud Storage ▪	whether use cloud storage to store data	true/false
	Cloud Data Volume +	potential data volume used on cloud storage	<µ, σ> or <µ, min, max>
	Storage Ingress Traffic +	data transfer-in speed for cloud storage (Mbps)	<µ, σ> or <µ, min, max>
	Storage Egress Traffic +	data transfer-out speed for cloud storage (Mbps)	<µ, σ> or <µ, min, max>
	VM to Storage Traffic +	communication from machine to storage (Mbps)	<µ, σ> or <µ, min, max>
	Storage to VM Traffic +	communication from storage to machine (Mbps)	<µ, σ> or <µ, min, max>
	Data Durability *	reliability demand for data stored on cloud storage	durability type
**Payment Preferences/User specified**	Rental Time +	time period to rental cloud resources	[t1, t2]
	Fee Constraint +	acceptable maximum monetary cost	upperBound
	Billing Type *	Pay as you go/subscription/bidding/…	BillingType_ID

Three features of this table are relevant. First, the hardware requirements specify the qualified hardware configuration to run the application. For numeric parameters, the minimum and preferred configurations are required. Second, application features describe the software requirements and computational features. The numeric parameters are derived from statistical values (e.g., mean value or *µ*, standard deviation or *σ*, minimum value or *min* and maximum value or *max*) because of the inherent uncertainty and statistical features. Three, in payment preference, rental times are modeled as a time range (from minimum to maximum), while fee constraint is expressed as the acceptable maximum monetary cost.

The function of criteria in service classification model is service filtering. The parameters in the application requirement schema depict the application-specific requirements in detail. More parameters are quantitative and fine-grained. Based on the schema, the cloud solutions can be generated using qualified resource offerings (e.g., VM types, cloud storage types). Moreover, the schema provides essential information for potential rental fee prediction and analysis on computational intensity features.

### 3.5 Preference-Aware Solution Evaluation Mode

Cloud solution selection is a Multi-Objective Decision Making (MODM) [34] process [11], [12] and in which multiple criteria (e.g., fee, SLA, performance, customer service) are objectives. In selecting multiple objectives, there is always a trade-off as one objective may influence/compromise another (i.e., Pareto efficiency). No universal evaluation principle maximizes the satisfactions to all requirements. Moreover, individual users have different preferences for their objectives. Accordingly, we propose a preference-aware evaluation, in which users select options to interactively change the importance of other objectives according to their preferences; and importantly this interactive evaluation and ranking happens “on-the-fly”.

In this paper, six criteria for demonstrated objectives are selected as follows: fee cost, VM computing capacity, SLA, user feedback, customer services and software ecosystem. Fee cost is the potential monetary cost for cloud services, and VM computing capacity measures the computational capability of the adopted individual VM. The SLA is the service-level agreement announced by cloud providers, while user feedback collects user, experience-based evaluation information complementing third-party measurement based methods. Customer service measures the convenience and quality of customer service. And finally, software ecosystem measures the fertility of software products provided by the cloud or contributed by third parties and the maturity of the software resource market of the cloud provider. To further define this criteria, each is assigned a level of importance as follows: *Unimportant*, *Less important*, *Moderate*, *Important* and *Very Important*. To make sure the selected cloud solution is balanced with the multiple objectives, Eq. (1) is adopted:
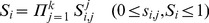
(1)


The *S_i_* is the synthesized cumulative score for solution *I* and *K* is the number of objectives. The *s_i,j_* is the normalized score of the *j*th objective for solution *I*, and *w_j_* is the importance of objective *j*. The predefined weights are 0, 0.3, 0.5, 1 and 2 for *Unimportant*, *Less important*, *Moderate*, *Important* and *Very Important*, in series. Given this equation, if all objectives are important, any one objective with a low score causes a low cumulative score for the solution. Conversely, if one objective is unimportant to the user, the score of this one objective does not affect the cumulative score. Therefore, this equation reflects user preferences on objective importance and helps balance the objectives. The scores of fee cost, VM computing capacity and user feedback for each solution are dynamically calculated in the solution evaluation stage, whereas the others are service level objectives calculated in advance. Specifically, the user feedback score is calculated by summarizing the collected grades (about involved cloud services and resources) from third-party cloud advisory systems. The SLA is collected from cloud providers' websites, and customer service is measured by counting the service channel types and collecting user feedback. The software ecosystem score is calculated by counting the type and number of provisioned software. The calculation of fee cost and VM computing capacity are discussed in the following subsections.

#### 3.5.1 Fee prediction

The ROI is a determining factor in cloud service/solution selection. To choose cost-effective solutions that meet users' budget constraints, a model is needed to predict monetary cost for each solution. Although variation exists on modes of resource offerings and billing for different cloud providers, a general model is proposed (Eq. 2):
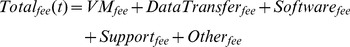
(2)


The model's parameters are defined as follows: total fee (Total*_fee_*) is a function of rental time *t*; VM*_fee_* is the fee spent on VMs tenancy; Storage*_fee_* is the fee for employing cloud storage or cloud databases (e.g., SQL Azure); DataTransfer*_fee_* is the potential service fee for data transfer; Support*_fee_* is the customer service fee; Software*_fee_* is the license fee or the fee charged for using extra software packages not included in basic software stack of a VM (e.g., Hadoop, SQL Server); Other*_fee_* includes fees for elastic IP address, extra network services (e.g., exclusive Content Delivery Network), and communication/processing/responding to different requests from others.

A cloud utilization fee is usually charged as a function of usage. The mutability of cloud usage may incur an uncertainty for the utilization fee, making it difficult to offer a precise prediction. To address this, a treat fee is introduced as a mean value with an estimated range (from potential minimum to maximum fee). Using the statistical values in the application requirement model, a rational range (e.g., [*max{0, µ - 3 σ }, µ +3 σ*] or [*min*, *max*]) for each application feature parameter is defined. Upon the ranges, the potential cloud disk usage and data transfer volume are calculated, and, total range and mean fees are calculated by summing the different fee components.

Based on the total fee, fee scores are calculated (Eq. 3) and normalized (Eq. 4). 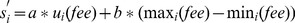
(3)




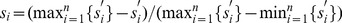
(4)


For a cloud solution, *u_i_(fee)*, *min_i_(fee)* and *max_i_(fee)* are the mean fee and two boundaries of the fee range in series, while *a* and *b* are weights. The *n* is the number of generated solution candidates. As the range interval is minimized, the predicted fee becomes more stable and mutability is reduced. Thus, the solution with a low mean cost and small range interval (low standard deviation) is a good solution.

#### 3.5.2 VM computational capability

The computational capability of a computing resource dictates its feasibility for individual applications. Multiple applications have a range of computational intensity features reflecting different hardware requirements.

To match the most suitable VM hardware configuration to the application features from numerous potential solutions, a computational capability measurement model is proposed based on the Technique for Order of Preference by Similarity to Ideal Solution (TOPSIS) [35], [36]. Six hardware indicators measure computational capability: *CPU core number, CPU speed, computing unit, RAM size, local disk size and bandwidth*. The first four are for computing performance. Local disk size measures VM storage capacity, whereas bandwidth measures network I/O performance.

The six indicators are normalized and weighed using Eq. (5):
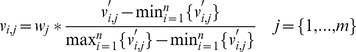
(5)


With 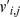 as the value of *j*th indicator for the *i*th cloud solution, *m* as the number of indicators, and w*_j_* as the importance of the indictor *j* for specific application (subject to). 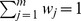
The weights are determined using Analytical Hierarchical Process (AHP) [12] and application intensity features. 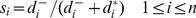
(6)


Since each of these selected indicators is positive (i.e., the higher value, the better the indicator), the positive ideal weighted value 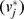 and negative ideal weighted value 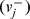 for indicator *j* are the largest and smallest weighted values in all solutions, respectively. The score *s_i_* of each cloud solution *i* is calculated using Eq. (6) by considering the distances to positive and negative ideal solutions, calculated using Eq. (7) and (8), respectively. 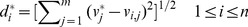
(7)




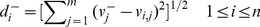
(8)


The higher the score value, the better the computational capabilities for a given application. The proposed model makes the computational capability of solutions quantitatively comparable. More specifically, the weighing mechanism introduces the indicator importance by considering different application scenarios, and the distances to positive and negative ideal solutions reflects the relative advantages and disadvantages on computing capacities of a solution in all solution candidates.

## Implementation and Experiments

### 4.1 System Architecture and Workflow

To implement, integrate and verify the proposed models, methods and technologies, the following architecture of cloud service recommendation system and recommendation workflow are proposed.

#### 4.1.1 Architecture

**Figure 6:**
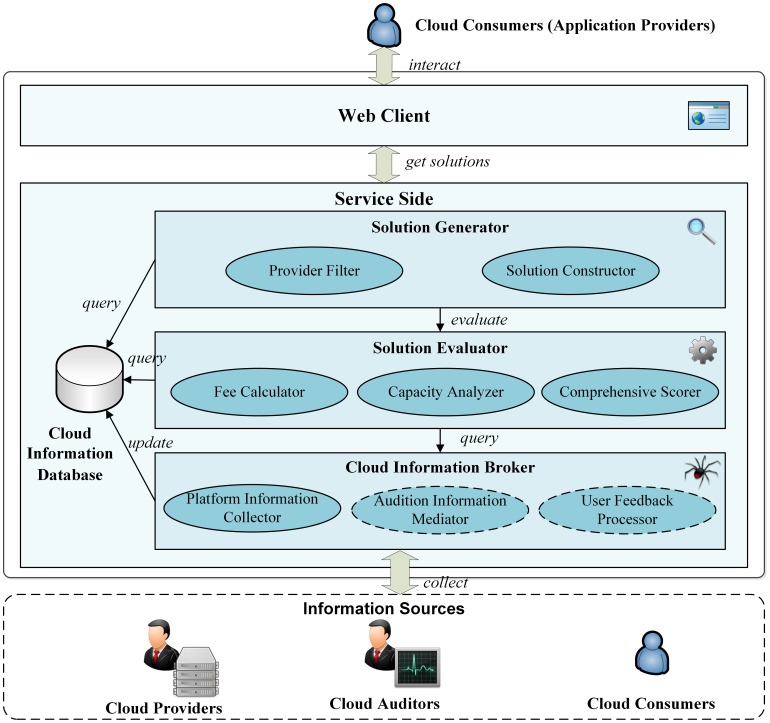
Architecture of cloud service recommendation system.

The architecture is based on the following components (Fig. 6):

The *Web Client* controls user interactions and provides solution presentation and visualization functions to assist in cloud solution selection. The server side includes four components: *Solution Generator*; S*olution Evaluator*; *Cloud Information Broker*; and *Cloud Information Database*. The *Solution Generator* generates feasible cloud solutions as a function of user inputs. It contains two sub-components: a *Service Filter* selects qualified cloud service from all service candidates according to service-level restrictions; and a *Solution Constructor* constructs feasible solutions based on application requirements and qualified services generated by the *Service Filter*. The S*olution Evaluator* assesses the suitability of the generated solutions according to application requirements and user's selection preferences. While a *Fee Calculator* calculates the potential fee cost, a *Capacity Analyzer* analyzes computational capacity. A *Comprehensive Scorer* grades and ranks solutions by counting multiple factors, and a *Cloud Information Broker* collects and integrates information from different sources and updates the *Cloud Information Database*. *Service Information Collector* collects information from cloud providers (see section 3.2). The *Audition Information Mediator* gathers and grades performance monitoring information from third-party auditors, and the *User Feedback Processor* collects feedbacks from cloud consumers (under development). Finally, the *Cloud Information Database* stores collected cloud information (e.g., pricing rules, configuration scheme, capability declarations, user feedbacks).

**Figure 7:**
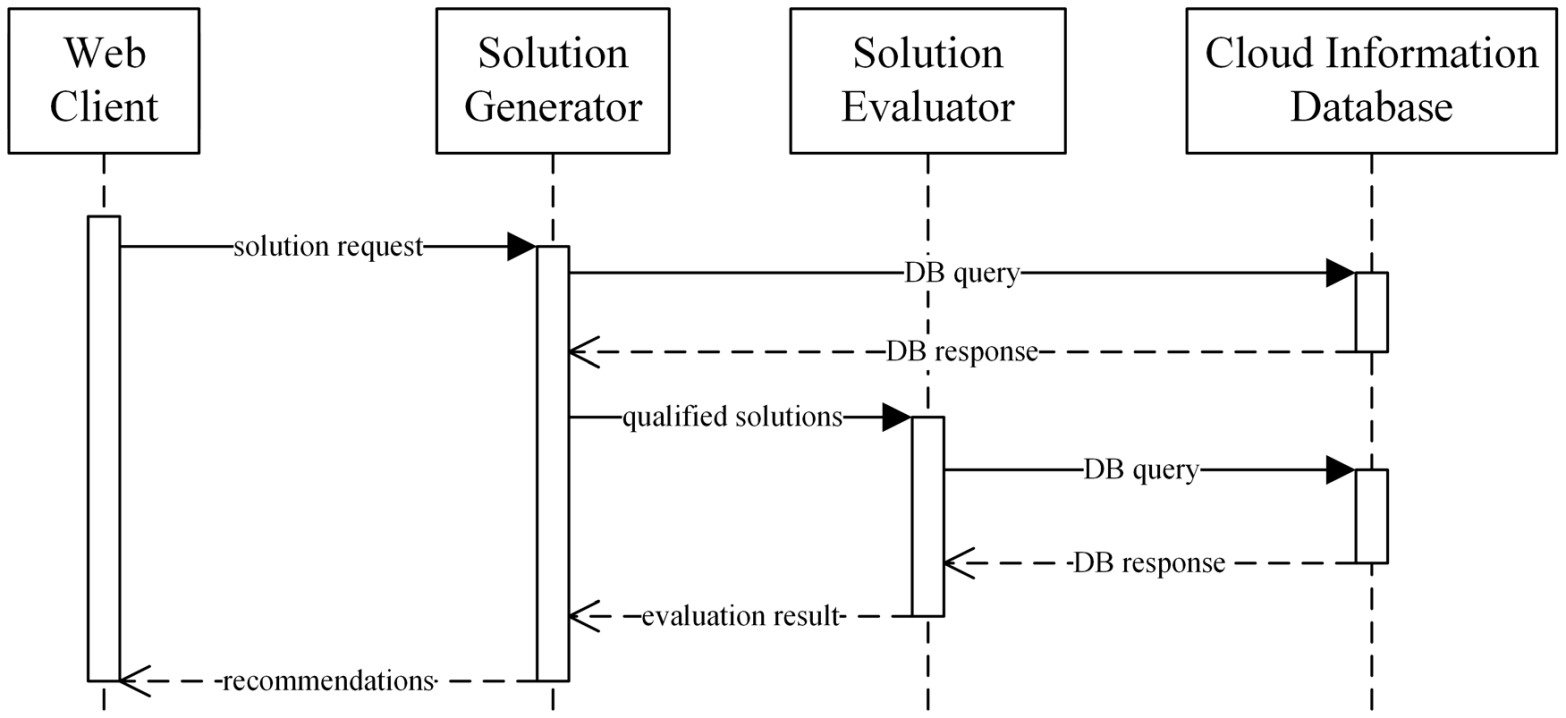
Interaction workflow of cloud service recommendation.

In the process of cloud service recommendation, *Web Client*, *Solution Generator*, *Solution Evaluator* and *Cloud Information Database* work collaboratively (Fig. 7). The *Cloud Information Broker* performs independently of this process. After user inputs are sent to the server side, the *Service Filter* is engaged, after which the *Solution Constructor* generates solutions with filtered services. When solutions are generated, *solution evaluator* evaluates all qualified solution. The *Fee calculator* and *Capacity Analyzer* work simultaneously (see section 3.5). Subsequently, the *Comprehensive scorer* ranks each solution by leveraging multiple criteria, including cost and VM capacity score.

#### 4.1.2 A four-phase recommendation workflow

**Figure 8:**
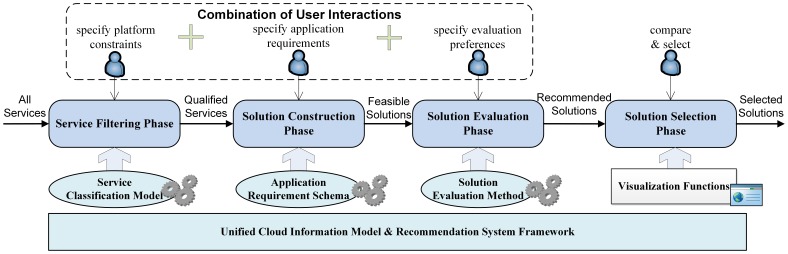
Workflow of four-phase recommendation framework.

With rational workflow, appropriate interaction and visualization technologies, the ease of using the solution evaluation and selection becomes commonplace. Based on this consideration, a four-phase recommendation workflow is proposed (Fig. 8).

The unified cloud information model and a system framework are the bases of the entire recommendation workflow. At the service filtering phase, the service classification model specifies the service level requirements and constraints/filters the cloud services. At the solution construction phase, application level requirements are described using the application requirement schema; these codify rules for generating solutions from qualified services. Subsequently, solutions are evaluated and recommended based on users' evaluation preferences. And in the final phase, the users compare solutions through a visualization-enhanced web GUI.

### 4.2 Web GUI Design and Visualization Functions

An intuitive, interactive, and straight-forward graphic user interface (GUI) is essential for better cloud solution selection. A well-designed GUI improves user experience and helps convey important information to assist decision-making [37]. This is especially true for the web-based applications [38], [39]. In this prototype, the Dojo toolkit (http://dojotoolkit.org/) and widgets are selected to define the GUI and layout, while Google Maps/Charts provide visualization and interaction function.

#### 4.2.1 Categorizing requirements input panels

**Figure 9:**
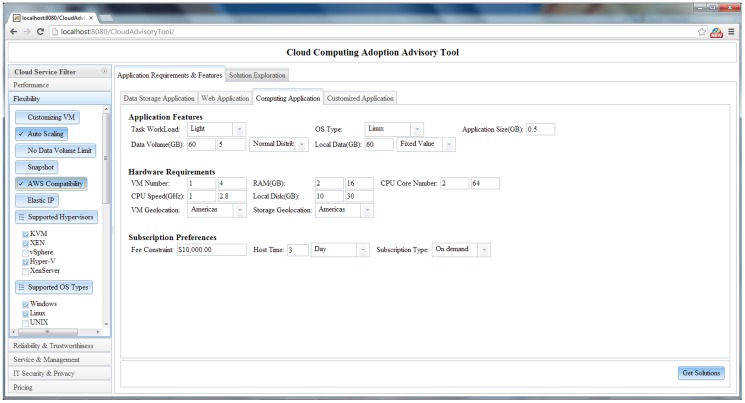
GUI for specifying application & service requirement.

To conduct a reliable evaluation, comprehensive requirements and preferences on applications and cloud services are essential. However, the process of complicated parameter input frustrates many users. User-friendly GUI, input wizards and tooltips simplify this process. An expandable service filtering panel - “Cloud Service Filter” (the left side panel in Fig. 9) allows experts to customize service level demands and constraints. Four application requirement input panels (tabs on the center of Fig. 9) are defined according to application types, as the same application type may have similar requirements. The *Data Storage Application Tab* stores simple cloud-based data where no application needs to be deployed. The *Web Application Tab* is for small-to-medium scale web applications, such as geospatial web portals and web services (e.g., web map services). The *Computing Application Tab* is for computing intensive requirements (e.g., dust storm forecasting, [22]). For experts who have a sufficient knowledge of the features of target application and cloud computing, the *Customized Application Tab* is a panel to specify elaborate requirements and from which the system can generate more reliable evaluations and recommendations. The value input parameters are predefined and dynamically adjusted according to the application type and user inputs in the tabs.

#### 4.2.2 Visualization and interaction for selecting cloud solutions

**Figure 10:**
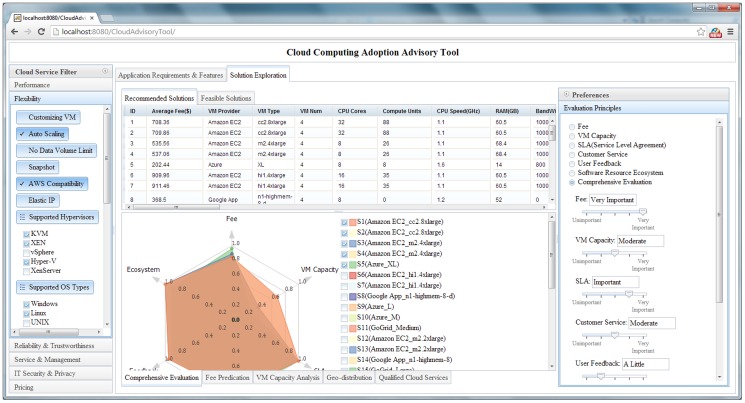
GUI for cloud solution exploration.

**Figure 11:**
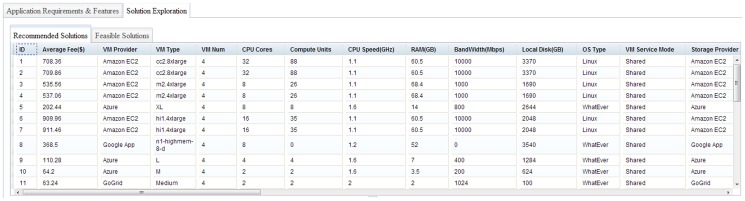
Data tables for recommended solutions and all feasible solutions.

**Figure 12:**
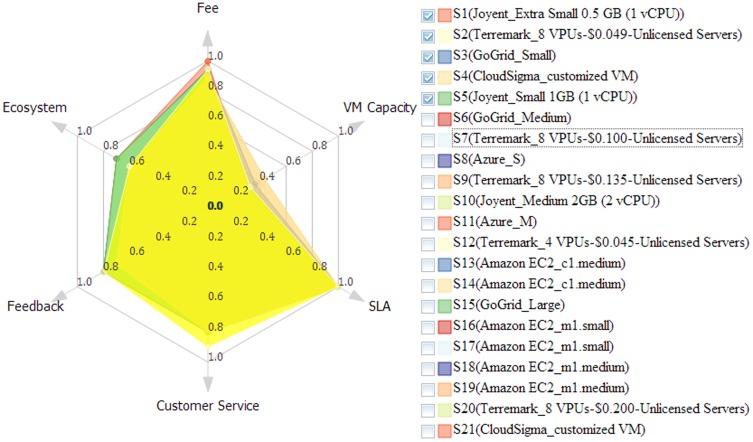
Rader chart for comprehensive evaluation of recommended solutions.

**Figure 13:**
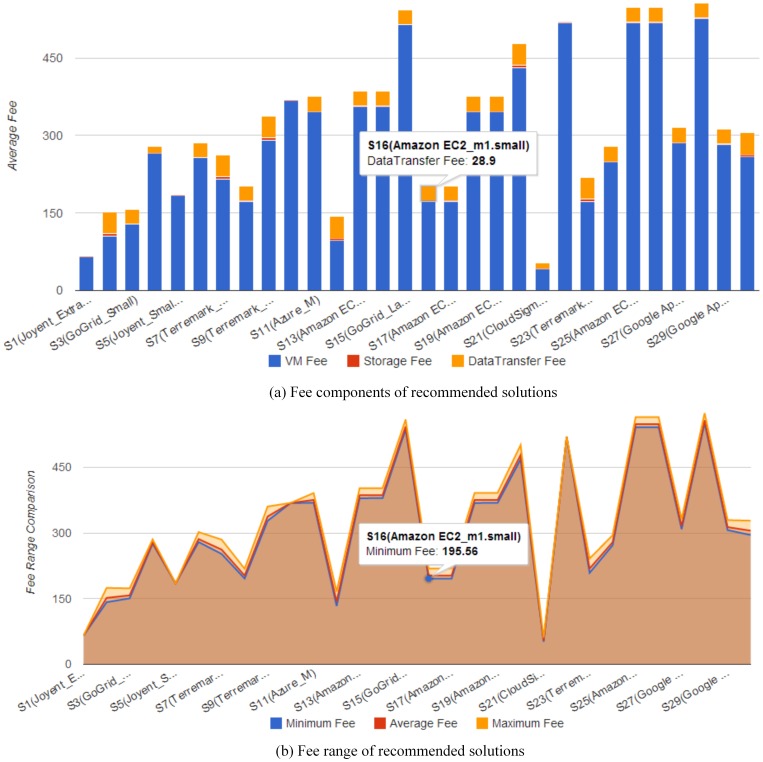
Potential fee ranges of recommended solutions: (a) fee components of recommended solutions; (b) fee range of recommended solutions.

**Figure 14:**
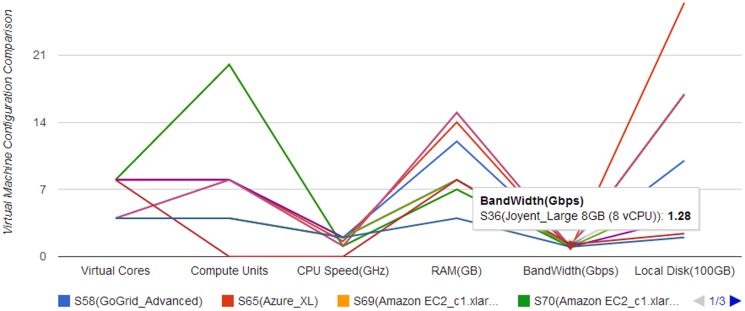
Virtual machine hardware configuration for recommended solutions.

**Figure 15:**
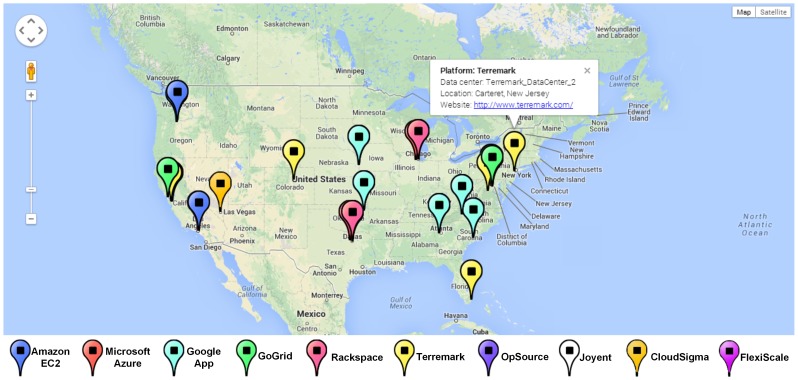
Data center distribution of cloud services.

**Figure 16:**
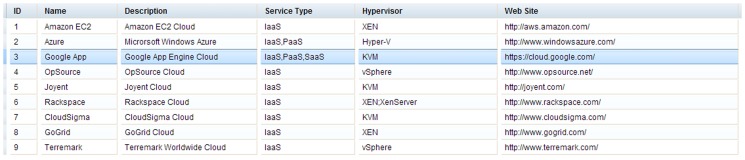
Data table of qualified cloud services.

To intuitively present and compare cloud solutions, multiple visualization and interactive methods are nested within the solution exploration GUI (Fig. 10), and these have six features. The first is data tables to exhibit solutions in detail (e.g., configurations, potential fee components, scores for objectives). The table *Recommended Solution* lists the solutions, whereas the *Feasible Solution* identifies all filtered feasible solutions (Fig. 11). A column-based re-sorting function allows one to compare solutions based on a variety of attributes. The second feature is the Comprehensive Evaluation Radar Chart (Fig. 12), which reveals the advantages and disadvantages of each solution by tabulating scores of evaluation objectives for the user-selected solutions. The third feature - Fee Charts (Average/Minimum/Maximum fee chart and fee range comparison chart) - (Fig. 13) compares potential fee ranges of for each solution. By visualizing the major fee components (e.g., VM fee, storage fee. data transfer fee), the users intuitively understands the percentages that each part costs. The fourth feature is the (line series based) Virtual Machine Configuration Chart (Fig. 14) in which one can compare VM computational capability parameters. The fifth feature - Geo-Distribution Maps (Fig. 15) - illustrates the geographical distribution of computing infrastructures (e.g., data centers) and potential end users of target applications using a map context. The geo-location information helps providers leverage spatial factors of resource allocation for cloud service selection. The sixth and final feature is the table of qualified Cloud services (Fig. 16), which is a brief description of general information of the selected services used in generating solutions.

Interactions are designed to make these visualization methods intuitive. Evaluating and ranking solutions “on-the-fly” allow users to adjust evaluation preferences at any time using the evaluation preference panel (right side panel, Fig. 10), which updates the solution tables and charts Moreover, users can specify the solution number and specific comparing solutions in charts. Finally, users can interact in data visualization either in tables or maps. Selecting a solution from the solution table, the data centers of the solution are highlighted. After the data center is clicked and the window displays the map, a brief introduction of the data center is offered and provides the URL of the cloud provider's website. Through the URL, users check the details and contact the cloud provider.

By leveraging the advancements of visualization technologies and web GUI design, the presentation and comparison of cloud solutions emerges as an intuitive feature for better supporting selection. Meanwhile, the user interaction becomes simple and efficient. Comparing many contemporary cloud advisory systems, the major feature of proposed prototype is its application-specified configuration of solutions and methods to help visualize, compare and evaluate solutions. Besides the map-based cloud infrastructure visualization function (Data Center Map) and the service features and user preferences based comparison functions (adopted by FindTheBest), the proposed prototype offers a preference-aware comprehensive evaluation function so users can specify their preferences “on-the-fly”. Potential usage fee predication and computational capability measurement functions are also integrated. In contrast to developing a desktop-based simulation system to analyze the frameworks and codes of target applications (e.g., CloudMIG), a web-based advisory system is proposed. This system recommends cloud solutions by leveraging user-specified application requirements, constraints and real-world cloud service provisioning.

### 4.3 Feasibilities of the Proposed Methods

#### 4.3.1 Effectiveness of service filtering method

From ten mainstream public cloud services their provisioning and pricing information were collected using the proposed cloud information broker. These services differ on business scales and adopt a range of technologies/strategies in cloud computing (Table 2).

**Table 2:** Selected features of the ten public cloud services.

Service Name\Features	HPC/GPU Cluster	Dedicated Server	VM Customization	Data Encryption	Global Data Center	Web-based Control Interface	Disaster Recovery	Minimum Billing Cycle	Auto-Scaling
Amazon EC2	X	X		X	X	X	X	1 h	X
Microsoft Azure	X			X	X	X	X	1 h	
Google App	X			X	X	X	X	1 min.	X
FlexiScale*			√					1 h	
OpSource		X	X		X	X	X	1 h	
Joyent		X		X	X			1 h	X
Rackspace		X		X	X	X	X	1 h	X
CloudSigma			X	X	X	X		5 min	
GoGrid		X			X	X		1 h	X
Terremark		X			X	X	X	1 h	X

*FlexiScale provides partial VM customization by re-sizing the memory and disk size.

Based on table 2, these services have disparate assets and capabilities. For example, distinct minimum billing cycles range from 1 min to 1 h. Three services (Google App, Amazon EC2 and Microsoft Azure) provide HPC support but currently only EC2 offers GPU Cluster. Three services support VM configuration customization function. Using the systematic service classification model and critical features, a qualified cloud service can be quickly identified. Subsequently, additional processes on cloud solution generation and evaluation handled by other modules will become efficient.

#### 4.3.2 Requirement-driven solution generation

**Figure 17:**
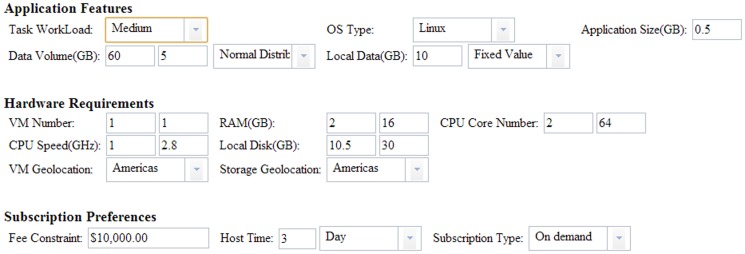
Requirements for demonstrated computing application.

The application requirement description schema guarantees the generated solutions taking into consideration hardware requirements, OS types and geo-location restriction. Taking the following computing application (with medium workload) scenario as an example (Fig. 17), the minimum hardware configuration per task is a machine with >2 GB RAM and 2 CPU core. The application runs on Linux and needs 0.5 GB local disk. Furthermore, the task requires the VM to have >10 GB local disk to host local data and ∼60 GB cloud storage to inventory the final result. The Americas is the geo-location restriction on VM and cloud storage. The user wants to host and run the task for 3 days.

According to the requirements, 94 feasible cloud solutions from nine cloud service providers (excluding FlexiScale which does not have a cloud infrastructure in Americas) are generated. If the minimum hardware configuration is changed to 16 GB RAM and 8-core, 43 solutions remain, less than half of the original. If the user changes the geo-location of VM and storage to Asia, 27 solutions from five vendors (EC2, Azure, Google App, Rackspace and Terremark) are generated. Solution generation is also affected by the parameters of computational features. For example, concurrent access intensity of a web application determines not only the network configurations of a cloud solution but even the instance number or scaling strategies. The task workload of a computing application can determine the hardware configuration of VMs.

#### 4.3.3. Preference-sensitive solution evaluation

**Figure 18:**
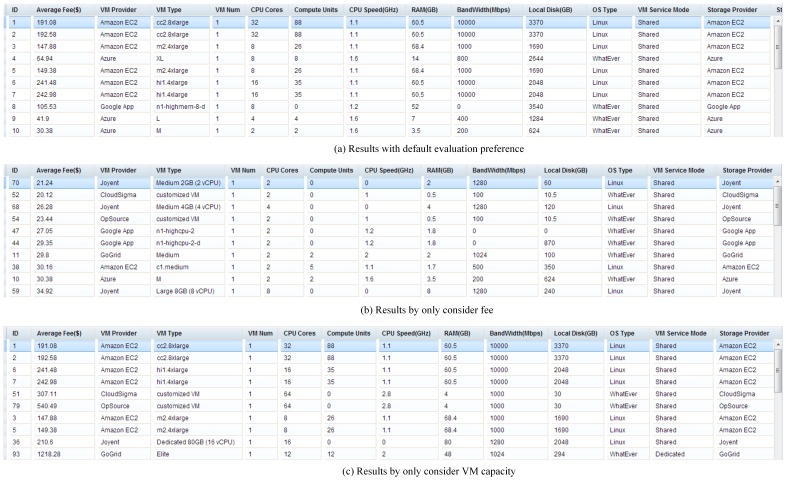
Recommended solutions with different evaluation preferences: (a) results with default evaluation preference; (b) results by only considering fee; (c) results by only considering VM capacity.

Solutions are dynamically assessed and ranked according to the importance of evaluation objectives. Therefore, the recommended solutions reflect the users' preferences. This approach is applied to a real cloud solution and the first ten recommended solutions for three different preferences are illustrated (Fig. 18) using the computing application scenario. The results with default preference (i.e., importance of fee, VM capacity, SLA, customer service, user feedback software resource ecosystem set as “very important”, “moderate”, “important”, “moderate”, “a little” and “unimportant”, in series) (Fig. 18a). The same analysis but including a fee is illustrated in Fig. 18 (b) while the results with the inclusion of a VM capacity are shown in Fig. 18 (c).

In summary, this dynamic and interactive ability to test the role of user's preference “on the fly” is a significant advancement to help users explore more effectively their assumption and thus accelerate their research initiatives by considering different priorities.

#### 4.3.4 Accuracy of fee prediction

Fee prediction is critical to end users as significant deviation on fee prediction dramatically affects the decision making on cloud solution selection. To validate the utility of the proposed method, the practical cloud usage and fee cost of a global accessible web application, GEOSS Clearinghouse (CLH) [40], on Amazon EC2 were analyzed. The application is a core component of Global Earth Observation System of Systems (GEOSS) Common Infrastructure for supporting geospatial data discovery and utilization. By 06 May 2013, 105 catalogues and 125,000metadata have been registered in the CLH and shared among 140 countries [41]. The application uses an m1.large Linux instance in the US East region and adopts a pay-as-you-go billing. The monthly cloud usage of CLH from July 2011 to October 2012 is summarized by analyzing log files and Amazon billing statements (Table 3). From this information, predictions can be offered of the monthly fee cost from November 2012 to April 2013 (6 months).

**Table 3:** Monthly cloud usage of CLH (July 2011– October 2012).

Features	Mean	Standard Deviation	Minimum	Maximum
CPU h per day (h)	24	0	24	24
EBS data size (GB)	193.305	35.349	160	287.325
EBS I/O requests	53650312.19	100360034	102942	307766613
EBS snapshot size (GB)	3.727	0.038	3.609	3.739
Data In-Transfer (GB)	17.146	28.534	1.321	111.451
Data Out-Transfer (GB)	20.407	48.026	0.213	204.174
Data Transfer (Regional) (GB)	5.507	6.738	0.147	27.143

**Figure 19:**
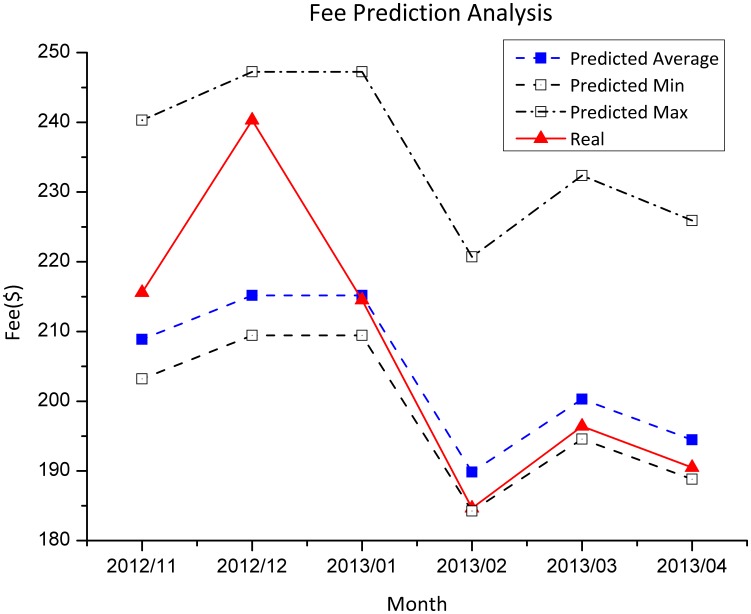
Monthly cloud usage fee predication analysis for CLH in EC2.

The predicted results (Fig. 19) show that the monthly average prediction error and standard deviation between real fee and predicated mean fee was 3.46% and 3.57%, respectively. December 2012 had largest predication error because that month had extreme EBS I/O requests (305,235,693). The range between predicted minimum and maximum fee and most of the errors were introduced by uncertainty of data transfer, storage volume and I/O operations, whereas the prediction on VM fee is relatively accurate since the CPU hours were stable. Therefore, if cloud usage is relatively stable, prediction the fee will be accurate. This is especially true for the applications which do not involve spatiotemporal dynamics on cloud usages. For web applications, the changes on user access intensities may dramatically impact fee prediction due to the changes in data transfer, storage and VM usages. Auto scaling may also influence fee prediction by introducing uncertainty on used CPU hours. Therefore, it is difficult to predict with precision the short-time (e.g., days) behavior and fee. However, for a long-time tenancy, the proposed model provides acceptable predictive estimates of fees. Furthermore, analyzing the long-time behaviours and spatiotemporal dynamics of the target application, the patterns of application feature parameters can be obtained. Based on that, the fee model may produce more reliable predictions.

## Conclusions and Future Work

This paper introduces a comprehensive framework and associated methodologies for brokering and recommending cloud services for application owners. Instead of only providing capabilities to compare the functional/non-functional properties, cost and ranking of cloud services at service level (coarse-granule), a novel method is proposed to generate, evaluate and recommend cloud service at the level of solution configuration (fine-granule). Four major conclusions are offered from this research.

First, To unify and integrate heterogeneous and multi-source cloud information from different cloud providers, auditors and consumers, a unified cloud information model is proposed. This model describes different cloud information and their relationships including service offerings, pricings, infrastructure distributions, basic features and capabilities, user feedback, and auditing.

Second, to categorize and refine user requirements and preferences for better supporting solution generation, an operational service classification model and an application-dependent requirement description schema are proposed. The former filters cloud services based on service-level features and constraints, while the latter depicts hardware requirements, application features and subscription preferences in detail. The integration of the two models provides an approach to create a user-requirement-qualified cloud solution.

Third, to comprehensively and flexibly evaluate generated cloud solutions, a user preference-aware and dynamic changeable solution evaluation mode is proposed. A fee prediction and VM computing capacity measurement method are defined to support the proposed evaluation method. By using these methods, solution can be evaluated and sorted according to user's preferences based on multiple evaluation criteria.

Four, to validate the feasibility of the proposed methods, an architecture and prototype of cloud advisory system is introduced. This system collects and updates cloud information from cloud providers' website using the proposed collecting mechanism. The adopted web visualization and interaction technologies facilitate the integration, presentation and comparison of cloud information and solutions, and make these functions easily accessible to users.

To further improve and expand the proposed brokering and recommendation capabilities, the direction of future research should target the following:

### Integrating performance monitoring information

Third-party audition information can help the advisory system make more reliable recommendations by providing near real-time availability, performance status and grades from long-time monitoring and analysis. Therefore, the mechanism to broker/collect, manage and utilize the performance information in the recommendation workflow should be investigated.

### Investigating a cloud information update mechanism and specifications

The web page parsing-based collecting method inevitably introduces unwanted issues such as timeliness. The recommendation system cannot guarantee all gathered provisioning information reflects the latest conditions. Another issue is the expensive cost for parser maintenance since the URL and content of the web pages may frequently change. In the future, it is proposed that one utilize the notification mechanism by cloud providers to trigger collecting information in a timely manner. Furthermore, formal and semantic-aided web service specifications for describing and querying heterogeneous cloud information are emerging needs to improve interoperability and brokering capabilities.

### Improving solution generation strategies and evaluation methods

Currently, the proposed system only provides static configured solutions (i.e., without considering scaling-up and scaling-down scenarios). To create solutions with dynamically changeable configuration strategies for different scenarios, more studies and experiments are required on real applications to learn their computational characteristics, spatiotemporal dynamics and requirements. This may also result in collaborate with third-party tools (e.g., CloudSim) to simulate cloud resource provisioning and consumption.

### Creating solutions for private and hybrid clouds

Private and hybrid cloud services, which provide more freedom and privacy of open cloud, are two major features of cloud computing. Therefore, evaluating cloud solutions with open-source cloud management software and private/hybrid cloud environments is needed. This should investigate relevant evaluation mechanisms and methodologies, and a means to integrate them into the proposed recommendation framework.
